# Supplementary material for: Crash dynamics of interdependent networks
Source: Sci Rep. 2019 Oct 10;9:14574. doi: 10.1038/s41598-019-51030-1 (PMC6787334; doi:10.1038/s41598-019-51030-1)
Supplement: Supplementary file 1 — Supplementary Information [file 41598_2019_51030_MOESM1_ESM.pdf]

Crash dynamics of interdependent networks
Supplementary Information

Jie Li[1,2], Chengyi Xia[1,2,*], Gaoxi Xiao[3,4,*], Yamir Moreno[5,6,7]

[1]Tianjin Key Laboratory of Intelligence Computing and Novel Software Technology, Tianjin University of Technology, Tianjin 300384, China

[2]Key Laboratory of Computer Vision and System (Ministry of Education), Tianjin University of Technology, Tianjin 300384, China

[3]School of Electrical and Electronic Engineering, Nanyang Technological University, Singapore 639798

[4]Complexity Institute, Nanyang Technological University, Singapore 639798

[5]Instituto de Biocomputación y Física de Sistemas Complejos (BIFI), Universidad de Zaragoza, Zaragoza 50018, Spain

[6]Departamento de Física Teórica, Facultad de Ciencias, Universidad de Zaragoza, Zaragoza 50009, Spain

[7]ISI Foundation, Turin, Italy

[*]xialooking@163.com, EGXXiao@ntu.edu.sg

List of Supplementary Figures

Figure 1. Fraction of nodal pairs being remained within the whole network, where two-layered networks have the same topology type and nodal mapping relationship is assumed to be random. The two-layered networks are assumed as ER random, exponential and scale-free networks, respectively.

Figure 2. Fraction of nodal pairs being remained within the whole network, where two-layered networks have the same topology type and nodal mapping relationship is assumed to be disassortative. The two-layered networks are assumed as ER random, exponential and scale-free networks, respectively.

Figure 3. Fraction of nodal pairs being remained within the whole network, where one network holds the exponential topology but the other one is the ER random network.

Figure 4. Fraction of nodal pairs being remained within the whole network, where one network holds the scale-free topology but the other one adopts the exponential topology.

Figure 5. Threshold q_{th} of modified KQ cascade under different detachment logic rule, in which the upper layer is the scale-free network but the lower one is a random graph.

Figure 6. Threshold q_{th} of modified KQ cascade under different detachment logic rule, in which the upper layer is the scale-free network but the lower one is an exponential network.

Figure 7. Threshold q_{th} of modified KQ cascade under different detachment logic rule, in which the upper layer is the exponential network but the lower one is a random graph.

Figure 8. Cascade size on the same type of complex networks with size $N = 10^4$, in which the nodal mapping mode between two-layered networks is assortative.

Figure 9. Cascade size on the same type of complex networks with size $N = 10^4$, in which the nodal mapping mode between two-layered networks is disassortative.

Figure 10. Cascade size on the different type of complex networks with size $N = 10^4$, in which the upper layer topology is set to be scale free and the lower layer topology is assumed to be exponential.

Figure 11. Cascade size on the different type of complex networks with size $N = 10^4$, in which the upper layer network is set to be an exponential network and the lower layer network is assumed to a random graph.

Fractions of remaining nodes

The mapping relationship among nodes on two-layered networks may also affect the evolutionary behavior of two interdependent networks, no matter what kind of logic is adopted. The remaining fraction of nodal pairs within two-layer networks under different detaching logic is shown in Fig.1 and Fig.2. Here, the nodal mapping relationship pattern is assumed to be random in Fig.1 or disassortative in Fig.2 between two networks, respectively.

Next, we focus on the evolution of fraction of remaining nodal pairs on interdependent networks with different topology type in Fig.3 and Fig.4. As a supplementary to the main text, we here hypothesize that a scale-free or ER random graph is adopted on one layer, but the other one is an exponential network. In Fig.3, we plot the fraction of remaining nodal pairs as a function of time steps under three kinds of coupling relationships between two networks, where the upper one is an exponential network and the lower one is an ER random graph. Meanwhile, the similar simulations are presented in Fig.4, but the network setup is set to be scale-free and exponential networks.

k_s-q_{th} curve and Cascade sizes

As shown in the Fig.5, Fig.6 and Fig.7, we illustrate the k_s-q_{th} curves of single networks and interdependent networks of various combinations including EXP-ER, SF-ER and SF-EXP under two different detachment rules. Additionally, we further present the evolution of cascade size by counting the fraction of remaining node pairs in the network after 500 time steps, and depict the cascade size upon

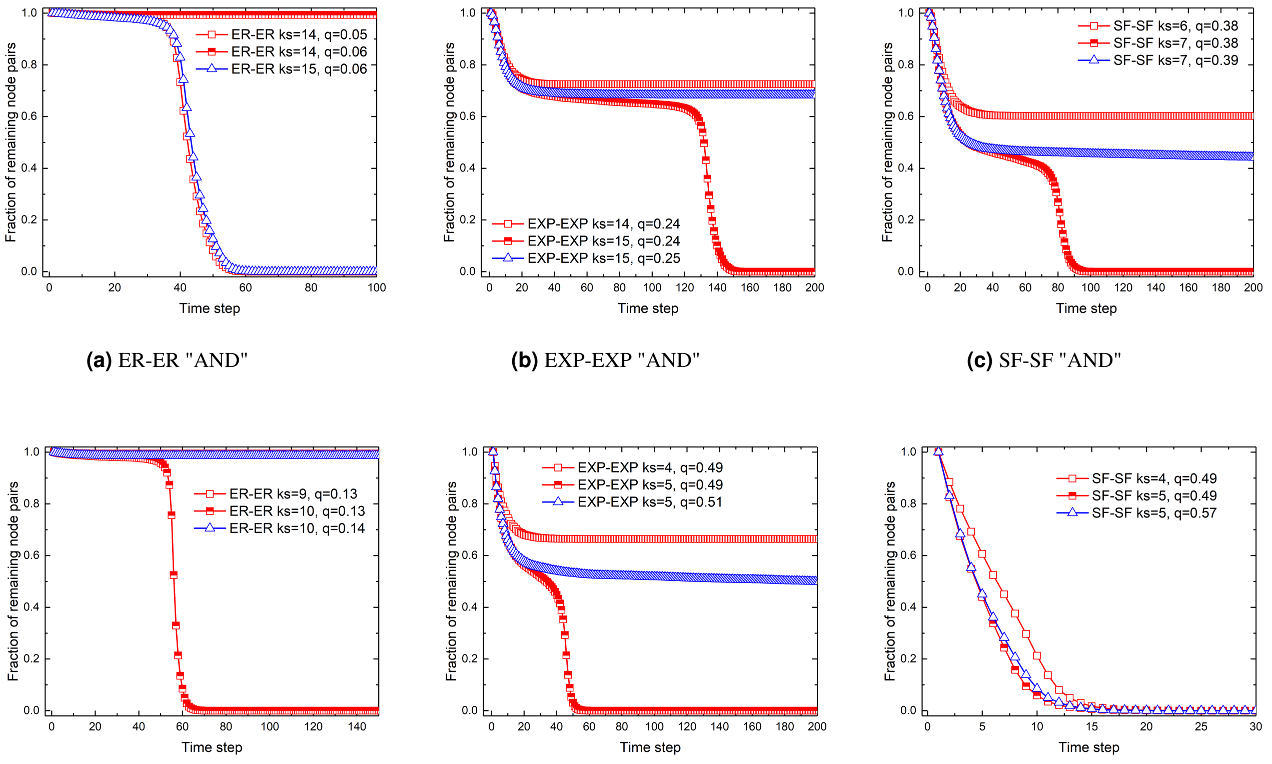

(a) ER-ER "AND" **(b)** EXP-EXP "AND" **(c)** SF-SF "AND"

(d) ER-ER "OR" **(e)** EXP-EXP "OR" **(f)** SF-SF "OR"

Figure 1. Fraction of nodal pairs being remained within the whole network, where two layered networks have the same type but different topology are assumed as ER random, exponential and scale-free networks, respectively; but the mapping relationship between two networks is supposed to be random. On the top three panels, the detachment logic for a nodal pair to leave the network is the "AND" logic, while the "OR" logic is adopted on the bottom three networks. Any node will be removed with the probability $f = 0.2$ once it fulfils the leaving condition according to the detachment logic. The network parameter setup is fixed to be $N = 10^4$, $< k >= 20$ for ER random graphs, exponential networks have the average degree $< k >= 20$ and a degree cutoff of 100 and scale-free ones take the power exponent $\gamma = 2$, the minimum degree of 3 and a degree cutoff of 100.

various types of networks for different values of k_s and q from Fig. 8 to Fig. 11. Among them, the mixing patterns among nodal pairs between two networks with the same network topology type are assumed to be assortative in Fig. 8 or disassortative in Fig. 9. Meanwhile, the evolution of the cascade size on two different networks is provided in Fig. 10, where one layer is a scale free network while the other one is an ER random graph, and results on two-layered networks with exponential and ER random setup are also given in Fig. 11.

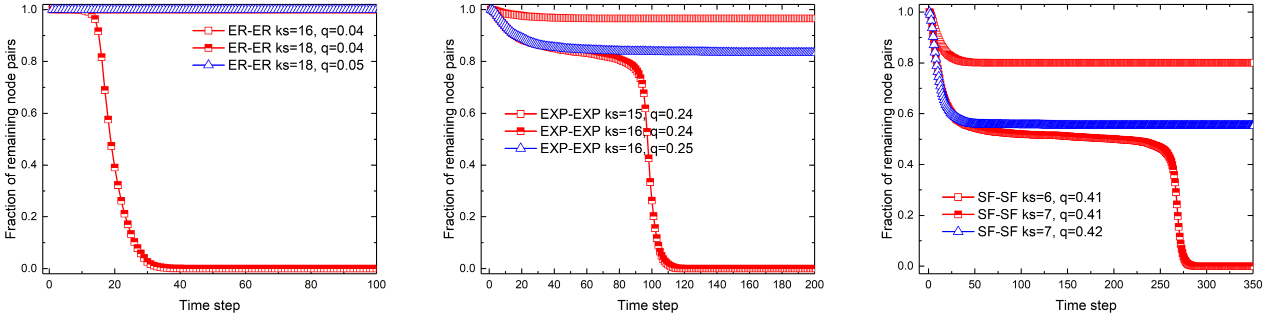

(a) ER-ER "AND" **(b)** EXP-EXP "AND" **(c)** SF-SF "AND"

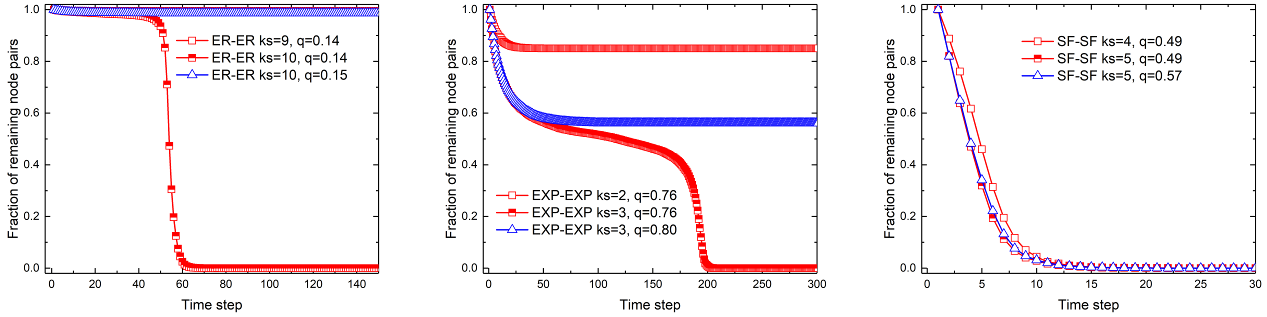

(d) ER-ER "OR" **(e)** EXP-EXP "OR" **(f)** SF-SF "OR"

Figure 2. Fraction of nodal pairs being remained within the whole network, where two layered networks have the same topology and are assumed as ER random, exponential and scale-free networks, respectively; but the current nodal mapping relationship is assumed to be disassortative. On the top three panels, the detachment logic for a nodal pair to leave the network is the "AND" logic, while the "OR" logic is adopted on the bottom three networks. Any node will be removed with the probability $f = 0.2$ once it fulfils the leaving condition according to the detachment logic. The network parameter setup is fixed to be $N = 10^4$, $< k >= 20$ for ER random graphs, exponential networks have the average degree $< k >= 20$ and a degree cutoff of 100 and scale-free ones take the power exponent $\gamma = 2$, the minimum degree of 3 and a degree cutoff of 100.

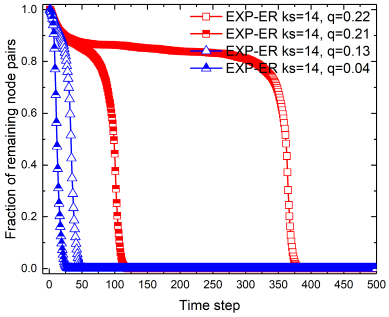

(a) EXP-ER Assortative "AND"

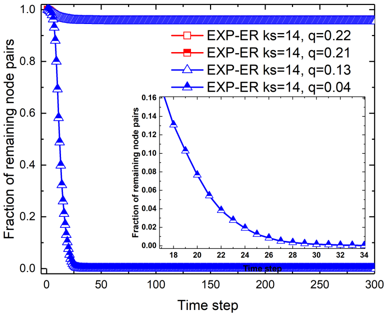

(b) EXP-ER Random "AND"

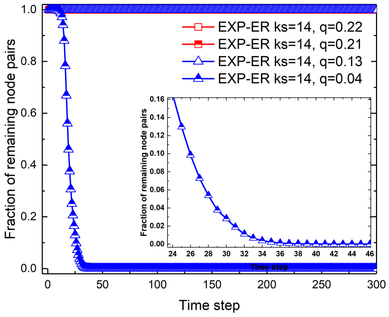

(c) EXP-ER Disassortative "AND"

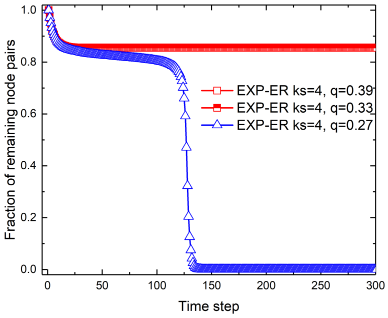

(d) EXP-ER Assortative "OR"

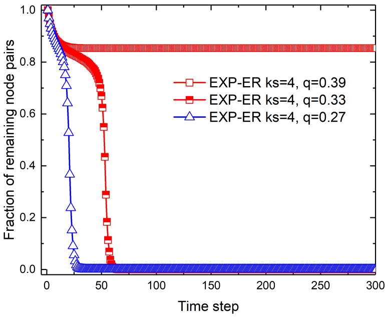

(e) EXP-ER Random "OR"

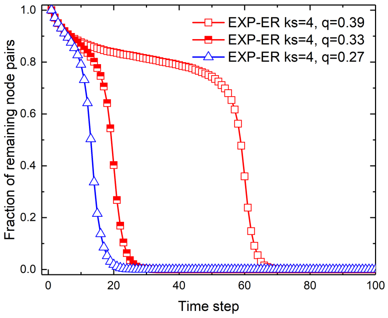

(f) EXP-ER Disassortative "OR"

Figure 3. Fraction of nodal pairs being remained within the whole network, where two layered networks are fixed to be exponential and ER random networks. On the top three panels, the detachment logic for a nodal pair to leave the network is the "AND" logic, while the "OR" logic is adopted on the bottom three panels. Any node will be removed with the probability $f = 0.2$ once it fulfils the leaving condition according to the detachment logic. In addition, the inset represents the enlarged area for the first 35 [panel (a) and (b)] and 50 time steps [panel (c)]. For all networks, the size is fixed to be $N = 10^4$, the average degree $< k >= 20$ for ER random graphs and exponential networks.

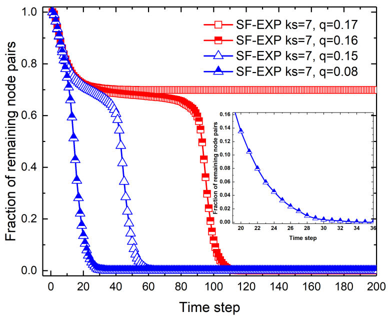

(a) SF-EXP Assortative "AND"

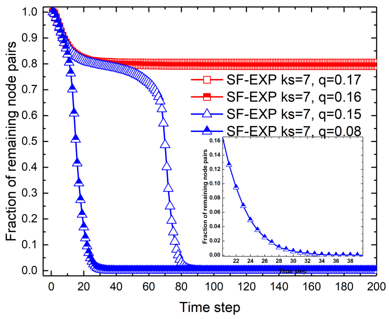

(b) SF-EXP Random "AND"

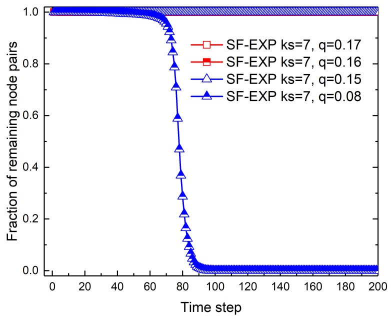

(c) SF-EXP Disassortative "AND"

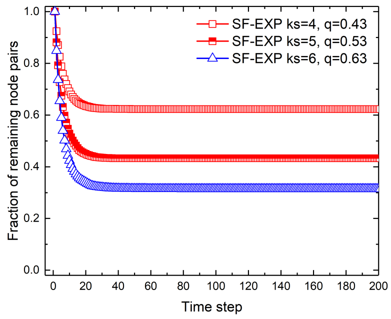

(d) SF-EXP Assortative "OR"

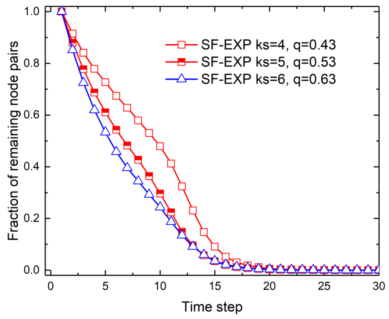

(e) SF-EXP Random "OR"

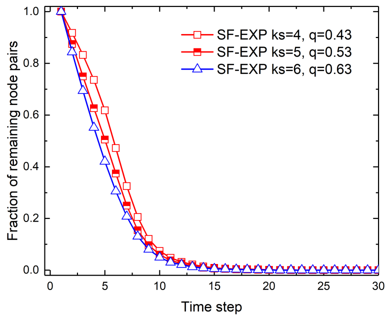

(f) SF-EXP Disassortative "OR"

Figure 4. Fraction of nodal pairs being remained within the whole network, where the two-layered networks are set to be scale-free and exponential networks. On the top three panels, the detachment logic for a nodal pair to leave the network is the "AND" logic, while the "OR" logic is adopted on the bottom three networks. Any node will be removed with the probability $f = 0.2$ once it fulfils the leaving condition according to the detachment logic. In addition, the inset represents the enlarged area for the first 35 [panel (a) and (b)] and 50 time steps [panel (c)]. For all networks, the size is fixed to be $N = 10^4$, and the average degree $< k >= 20$ for exponential networks, and scale-free ones take the power exponent $\gamma = 2$, the minimum degree of 3 and a degree cutoff of 100.

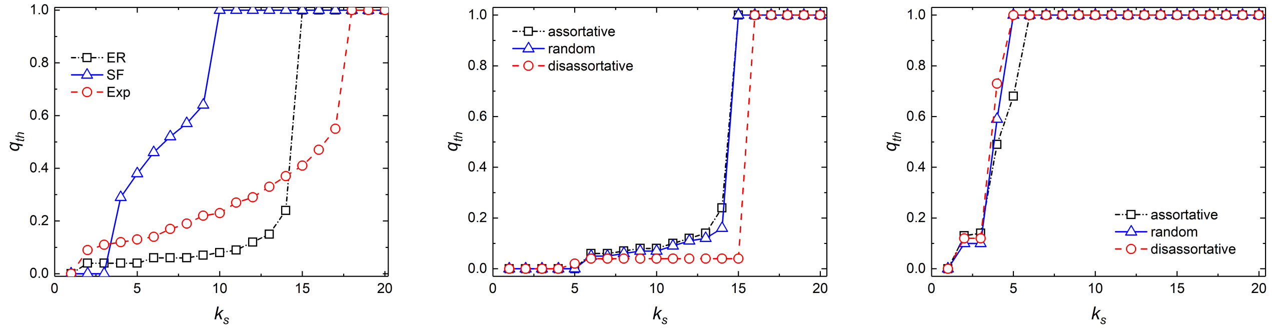

(a) single network **(b)** SF-ER "AND" **(c)** SF-ER "OR"

Figure 5. Threshold q_{th} of modified KQ cascade under different detachment logic rule, in which a trial-and-error is used to determine the threshold of q by increasing a step length of 0.01 for a specific k_s value, the two-layered networks are set to be scale-free and ER random networks. In the left panel (a), three different underlying topologies are set to be ER, Exp and SF networks with the network size $N = 10^4$; In the middle panel (b), the "AND" logic is used between scale-free network (upper layer) and ER network (lower layer), while the "OR" logic is used in the right panel (c). The same network structural parameters as those in Fig.1 are adopted from the panel (b) to (c).

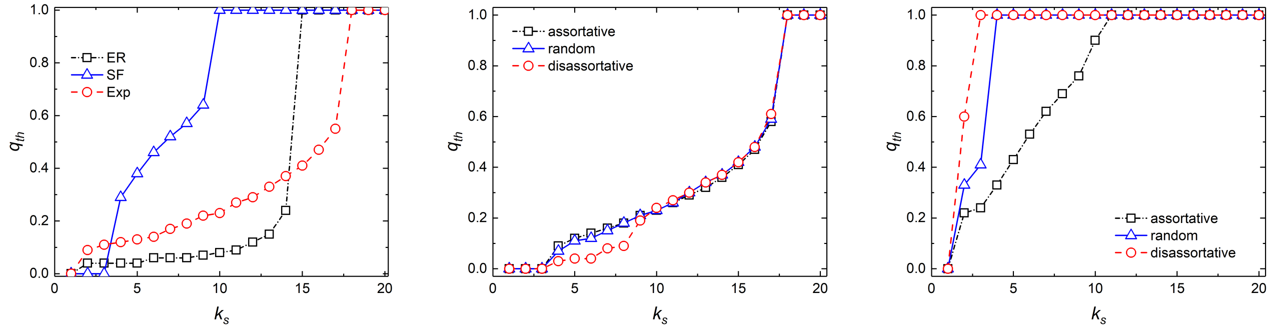

(a) single network **(b)** SF-EXP "AND" **(c)** SF-EXP "OR"

Figure 6. Threshold q_{th} of modified KQ cascade under different detachment logic rule, in which a trial-and-error is used to determine the threshold of q by increasing a step length of 0.01 for a specific k_s value, the two-layered networks are set to be scale-free and exponential networks. In the left panel (a), three different underlying topologies are set to be ER, Exp and SF networks with the network size $N = 10^4$; In the middle panel (b), the "AND" logic is used between scale-free network (upper layer) and ER network (lower layer), while the "OR" logic is used in the right panel (c). The same network structural parameters as those in Fig.1 are adopted from the panel (b) to (c).

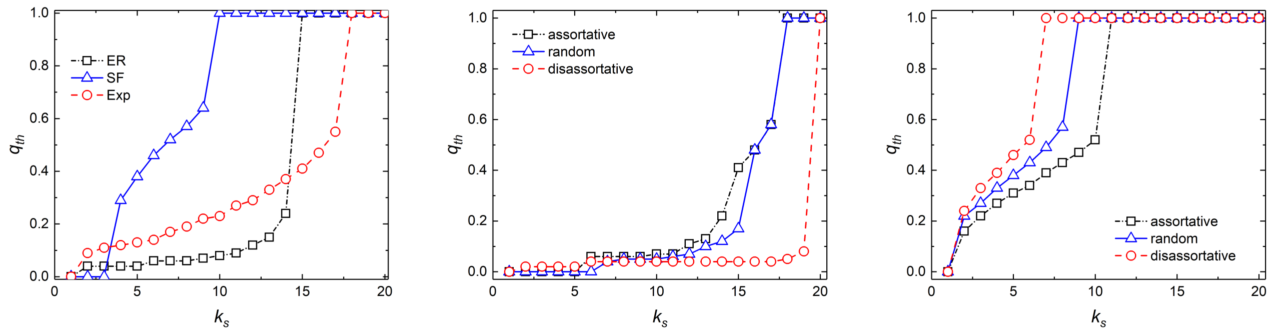

(a) single network **(b)** EXP-ER "AND" **(c)** EXP-ER "OR"

Figure 7. Threshold q_{th} of modified KQ cascade under different detachment logic rule, in which a trial-and-error is used to determine the threshold of q by increasing a step length of 0.01 for a specific k_s value, the two-layered networks are set to be exponential and ER random networks. In the left panel (a), three different underlying topologies are set to be ER, Exp and SF networks with the network size $N = 10^4$; In the middle panel (b), the "AND" logic is used between scale-free network (upper layer) and ER network (lower layer), while the "OR" logic is used in the right panel (c). The same network structural parameters as those in Fig.1 are adopted from the panel (b) to (c).

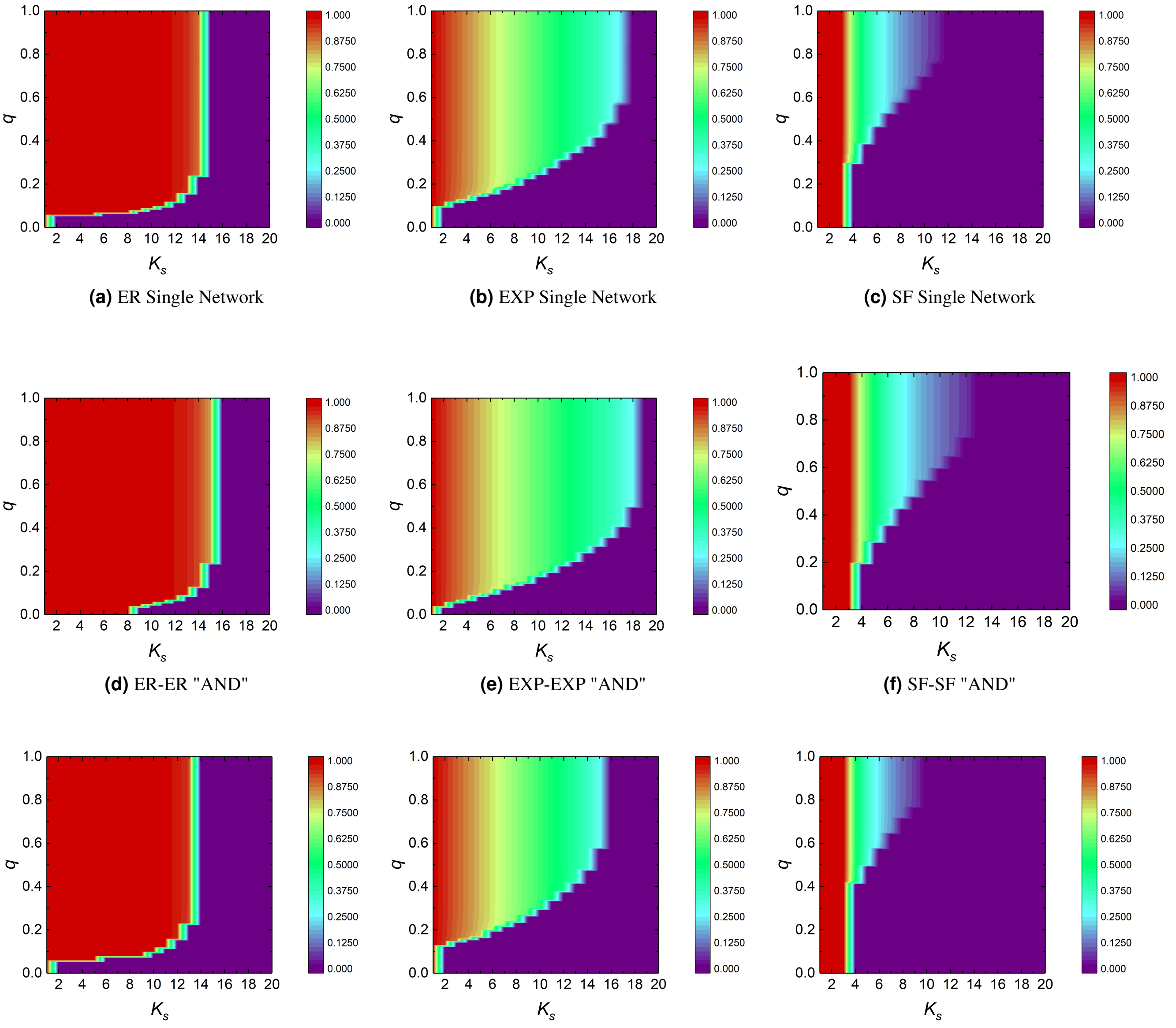

(a) ER Single Network
(b) EXP Single Network
(c) SF Single Network
(d) ER-ER "AND"
(e) EXP-EXP "AND"
(f) SF-SF "AND"
(g) ER-ER "OR"
(h) EXP-EXP "OR"
(i) SF-SF "OR"

Figure 8. Cascade size on different types of complex networks with size $N = 10^4$, in which the single network is adopted in the top three panels and two interdependent networks are assumed to hold the same topology type in the middle or bottom panels. From panel (a) to (c), the network topology is set as ER, exponential, scale-free ones, in which the standard KQ-cascade model is used to determine the cascade size; the middle three panels are two-layered interdependent networks in which the "AND" logic is adopted, while the "OR" rule is adopted in the bottom three panels. Meanwhile, from panel (d) to (i), the mapping relationship among nodes between two networks is set to be assortative. For all panels, the colour denotes the fraction of remaining nodal pairs within the network after 500 iterations. In addition, the matching pattern between two network is assumed to be random for the two-layered networks. All other parameters are set to be identical with those in Fig.1 and Fig.2.

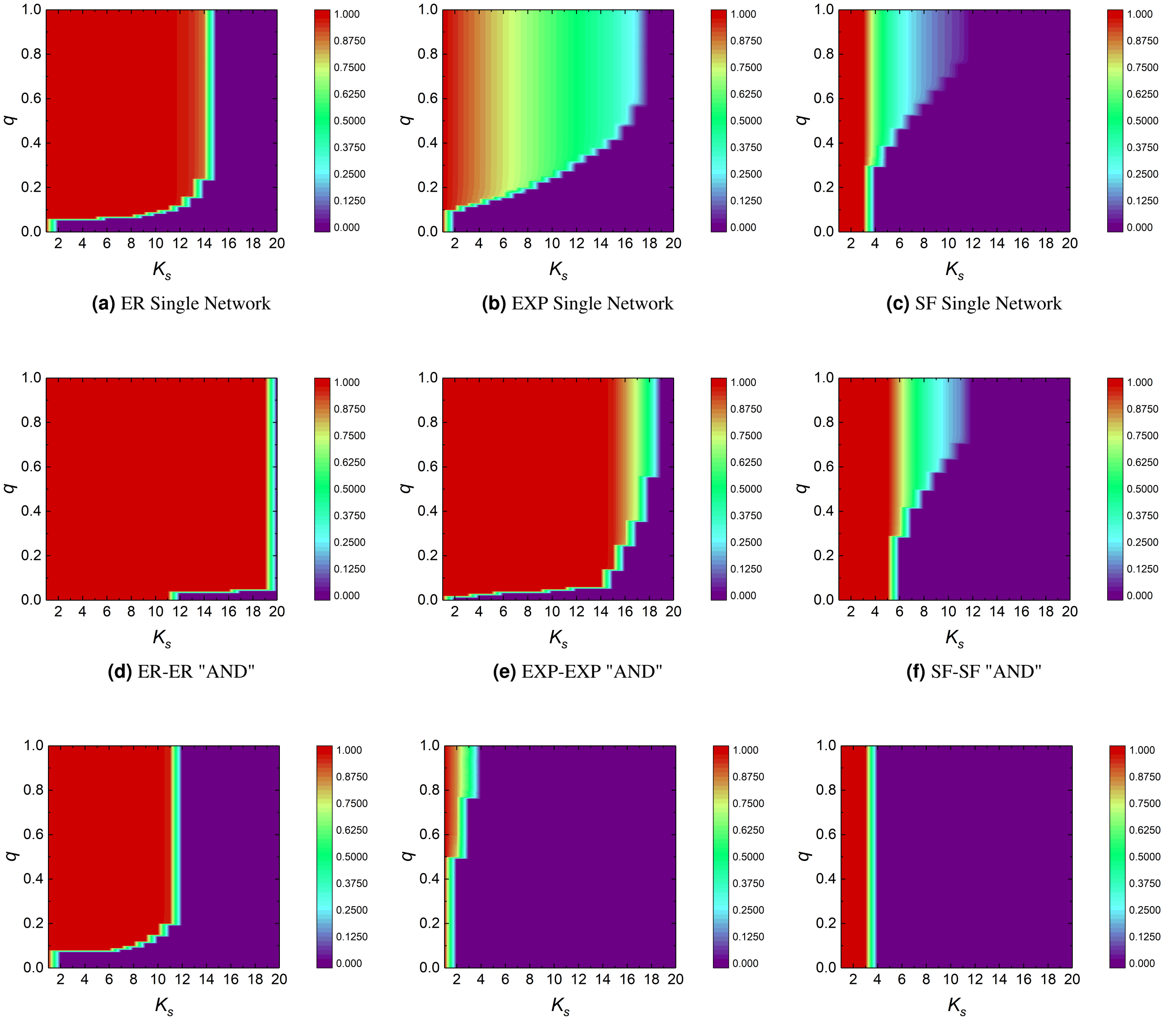

(a) ER Single Network **(b)** EXP Single Network **(c)** SF Single Network

(d) ER-ER "AND" **(e)** EXP-EXP "AND" **(f)** SF-SF "AND"

(g) ER-ER "OR" **(h)** EXP-EXP "OR" **(i)** SF-SF "OR"

Figure 9. Cascade size on different types of complex networks with size $N = 10^4$, in which the single network is adopted in the top three panels and two interdependent networks are assumed to hold the same topology type in the middle or bottom panels. From panel (a) to (c), the network topology is set as ER, exponential, scale-free ones, in which the standard KQ-cascade model is used to determine the cascade size; the middle three panels are two-layered interdependent networks in which the "AND" logic is adopted, while the "OR" rule is adopted in the bottom three panels. Meanwhile, from panel (d) to (i), the mapping relationship among nodes between two networks is set to be disassortative. For all panels, the colour denotes the fraction of remaining nodal pairs within the network after 500 iterations. In addition, the matching pattern between two network is assumed to be random for the two-layered networks. All other parameters are set to be identical with those in Fig.1 and Fig.2.

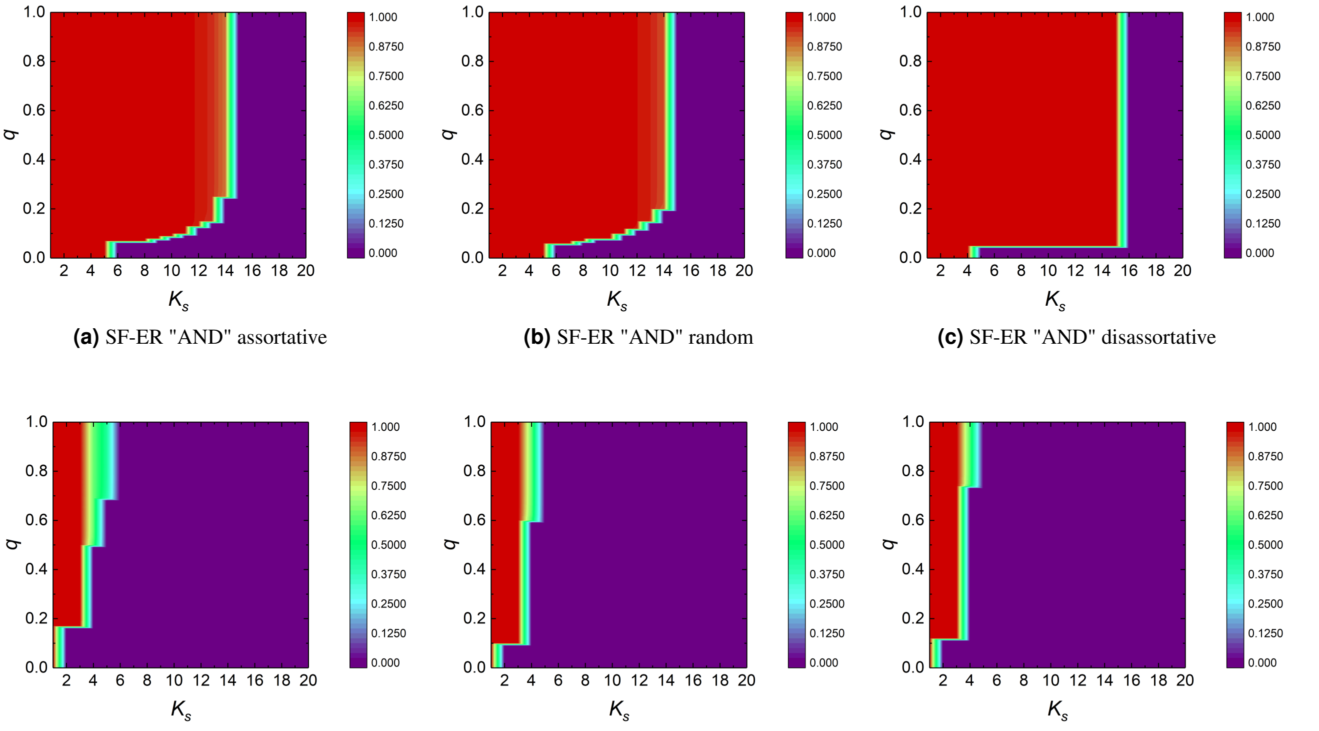

(a) SF-ER "AND" assortative **(b)** SF-ER "AND" random **(c)** SF-ER "AND" disassortative

(d) SF-ER "OR" assortative **(e)** SF-ER "OR" random **(f)** SF-ER "OR" disassortative

Figure 10. Cascade size on different types of complex networks with size $N = 10^4$, in which the upper layer topology is set to be scale free and the lower layer topology is assumed to be random. The top three panels provide the cascade size under the "AND" logic, while the bottom three panels give out the results for the "OR" logic. For all panels, the colour denotes the fraction of remaining nodal pairs within the network after 500 iterations. All other parameters are fixed to be same as those in Fig.3 and Fig.4.

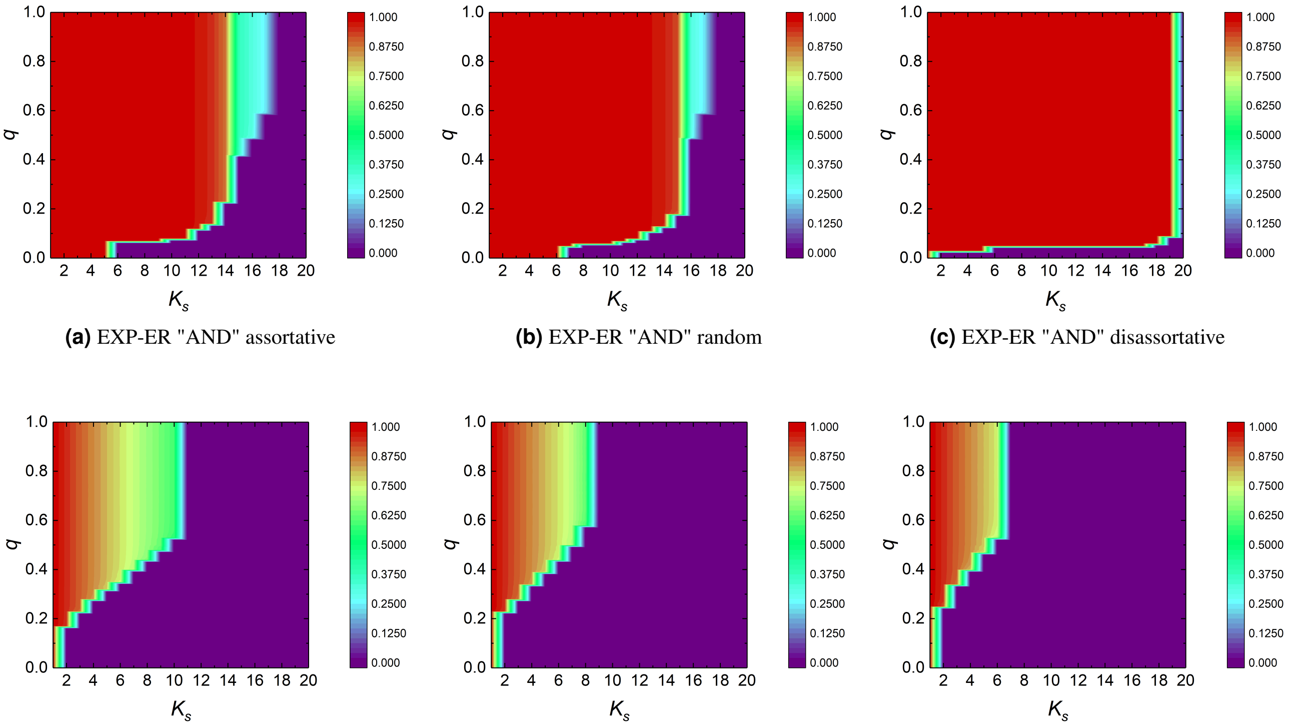

(a) EXP-ER "AND" assortative

(b) EXP-ER "AND" random

(c) EXP-ER "AND" disassortative

(d) EXP-ER "OR" assortative

(e) EXP-ER "OR" random

(f) EXP-ER "OR" disassortative

Figure 11. Cascade size on different types of complex networks with size $N = 10^4$, in which the upper layer topology is set to be exponetial and the lower layer topology is assumed to be random. The top three panels provide the cascade size under the "AND" logic, while the bottom three panels give out the results for the "OR" logic. For all panels, the colour denotes the fraction of remaining nodal pairs within the network after 500 iterations. All other parameters are fixed to be same as those in Fig.3 and Fig.4.
